# Effects of Neurosurgical Treatment and Severity of Head Injury on Cognitive Functioning, General Health and Incidence of Mental Disorders in Patients With Traumatic Brain Injury

**DOI:** 10.5812/atr.6546

**Published:** 2012-10-14

**Authors:** Sajjad Rezaei, Karim Asgari, Shahrokh Yousefzadeh, Heshmat-Allah Moosavi, Ehsan Kazemnejad

**Affiliations:** 1 Guilan Road Trauma Research Center, Department of Psychology, University of Isfahan, Isfahan, IR Iran; 2 Department of Psychology, University of Isfahan, Isfahan, IR Iran; 3 Guilan Road Trauma Research Center, Guilan University of Medical Sciences, Rasht, IR Iran; 4 Department of Psychiatry, Guilan University of Medical Sciences, Rasht, IR Iran

**Keywords:** Brain Injury, Neurosurgery, Cognitive Aspect, Mental Health

## Abstract

**Background:**

Neurosurgical treatment and the severity of head injury (HI) can have remarkable effect on patients’ neuropsychiatric outcomes.

**Objectives:**

This research aimed to study the effect of these factors on cognitive functioning, general health and incidence of mental disorders in patients with a traumatic brain injury (TBI).

**Patients and Methods:**

In this descriptive, longitudinal study, 206 TBI patients entered the study by consecutive sampling; they were then compared according to neurosurgery status and severity of their HI. Both groups underwent neurosurgical and psychological examinations. The mini mental state examination (MMSE) and general health questionnaire–28 items (GHQ-28) were administered to the study participants. At follow-up, four months later, the groups underwent a structured clinical interview by a psychiatrist based on the diagnostic and statistical manual of mental disorders, fourth edition (DSM-IV) diagnostic criteria regarding the presence of mental disorders.

**Results:**

Analysis of covariance (ANCOVA) and multivariate analysis of covariance (MANCOVA) were performed and adjusted for the effect of confounding variables (age, gender, Glasgow outcome scale (GOS) , and level of education). The severity of HI had the most significant effect for the following variables; cognitive functioning and physical symptoms (*P* < 0.05). The effect of the neurosurgical treatment factor was not significant; however, the interaction effect of the two variables on social dysfunction, and total score of the GHQ-28 questionnaire appeared to be significant (*P* < 0.05). Fisher's exact test indicated that after a four month follow-up period, no significant differences were seen between the two groups (with or without neurosurgery) in the incidence of mental disorders, while χ^2^ Test showed that having a more severe HI is significantly correlated with the incidence of mental disorders (*P* < 0.01).

**Conclusions:**

The implications of this study should be discussed with an emphasis on negative, effective factors on the cognitive – behavioral and neuropsychiatric outcomes of a TBI.

## 1. Background

Traumatic brain injury (TBI) is a catastrophic experience which changes individuals’ life after their injury and it is also one of the most important causes of death and long-lasting disabilities in 35-year-old and younger people. According to recent estimates, approximately 80to 90 thousand people live with prolonged disability following a TBI. Approximately 5% of those injuries led to the patient’s death and14% of these patients may remain in a moderate to severe state, while the remaining cases suffer from mild injuries (1, 2). These patients have residual impairments consisting of physical limitations, and also cognitive deficits, social limitations, and mental disorders (3-6). Meanwhile brain surgery for some patients with penetrating injuries and skull fractures may be indicated, which may be necessary to control their bleeding and to reduce intracranial pressure. No study has been yet reported about the effect of neurosurgical treatment on cognitive and psychopathological outcomes in TBI patients. However, Benedictus *et al*. (7) found that 40% of TBI patients had physical limitations, of which 62% showed cognitive complications and 55% had behavioral impairments. Cognitive impairment is usually defined as a deficit in the intellectual functioning which depends on: attention, information processing, language and memory (8). Cognitive deficits may be reduced within the first three months following a mild injury. However, some research studies have found that recovery from cognitive deficits may last much longer in mild TBI patients (9). Another point which emphasizes the importance of studying cognitive impairment in TBI patients, is the fact that they may become irritable, anxious, apathetic or depressed, due to cognitive deficits (10). In this regard, Landre *et al*. (11) have found that trauma patients with brain injuries, had scores worse than those for the trauma patients without a brain injury in all cognitive assessments. Finset *et al*. (12) have also revealed that deficits in patients with multiple trauma were related to the severity of the brain injury and the rate of psychological distress even three years after injury. Complications related to their health, have also been observed in most TBI patients and this may have a declining effect on their quality of life (13). Similarly, Heltemes *et al*. (14) have shown that self-rated levels of health after blast-related mild traumatic brain injury are significantly worse than that the trauma. The General Health Questionnaire-28 items (GHQ-28) are one of the most practical tools currently available for the evaluation of health levels. Using this test, Rezaei *et al*. (15) indicated that 90 TBI patients (n = 238) (58%) had serious problems in their general health and their GHQ scores were significantly correlated with a diagnosis of mental disorders, based on diagnostic and statistical manual of mental disorders, fourth edition (DSM-IV) diagnostic criteria. Furthermore, Nazari *et al*.(16) reported a significant relationship between the general health levels of brain damaged patients with the severity of their brain injury, depression and cognitive abilities. Nonetheless, it is generally postulated that the incidence of mental disorders and other neurobehavioral problems increase along with the severity of brain damage (17). The severity of the brain injury is usually determined in regard to scores of the Glasgow Coma Scale (GCS) which includes three categories; mild: 15 - 13, moderate: 12 - 9, severe: 8 and less (18). All of the studies that have been reported on the effects of brain injury severity in head trauma patients have emphasized the devastating role of a greater severity of TBI on psychopathological outcomes. For instance, Max *et al*. (19) demonstrated that TBI severity is the only factor by which personality changes following brain injury can be predicted. Two researches revealed that the severity of brain injury after TBI is related to the incidence of mental disorders (20) and prediction of suicidal behavior (21). Rezaei *et al*. (22) also reported that four months after a brain injury, the TBI severity was more evident in victims who had developed mental disorders, compared to those without mental disorders. There has also been some research on cognitive and behavioral outcomes of TBI, but there has been no well-established evidence about the effects of neurosurgical treatment and the severity of HI along with their interactive effects on: cognitive functioning, general health and the incidence rate of mental disorders in patients with TBI. Results of this present study could be useful in promoting our knowledge about the effects of neurosurgical treatment and the severity of brain injury on: cognitive status, general health and psychopathology of TBI patients, and this can be utilized to prepare neuropsychiatric rehabilitation protocols following a TBI.

## 2. Objectives

The main objective of this research was to investigate the effects of neurosurgical treatment and the severity of head injuries on: cognitive functioning, general health and the incidence of mental disorders in patients with TBI.

## 3. Patients and Methods

The statistical population of the present research included all TBI patients in the Guilan Province, Iran, in 2010. A total of 221 TBI patients were selected by non-probability and consecutive sampling methods and entered into a case-control study with four months follow-up. Although these patients may have been referred from the emergency, trauma and neurology wards of the Poursina hospital, legal medicine department in the Guilan province, or physicians from local clinics, the patients’ TBI diagnosis were all confirmed in a neurosurgery clinic by a neurosurgeon in the Imam Reza Specialized Clinic. Having satisfied inclusion criteria, each patient was then referred to a psychologist for psychological evaluations. Then the patient was referred to a psychiatrist for further examinations at least three months after the HI. The psychiatrist was one of the authors of this research, however, the information from the neurological evaluation, organic brain pathology and psychological assessments were blinded to him. It was hypothesized that having no information about the neurosurgical findings, could eliminate or reduce any non-blinded outcome assessment bias or diagnostic suspicion bias. Types of mental disorders were determined using structured clinical interviews by a psychiatrist based on DSM-IV diagnostic criteria with no mental disorder occurring merely in delirium and if there was dementia, no mental disorder could be assigned as a diagnosis. In addition, the disorder should not have been caused from another mental disorder (eg, substance abuse disorders). If a TBI patient was diagnosed with a mental disorder, then a clinical file was recorded for him to stay in therapy.

### 3.1. Inclusion Criteria

1) Age, 18 years or older, 2) Level of consciousness score < 15 based on GCS, a focal or diffuse injury of brain tissue due to an external mechanical force, 3) Loss of Consciousness (LOC) over 1 minute, 4) Post-traumatic amnesia (PTA) over 20 minutes, 5) Radiographic or CT findings showing TBI (eg, skull fracture, intracranial hemorrhage or acute brain abnormalities), 6) Headache, dizziness or nausea continuously for three days despite a GCS = 15.

### 3.2. Exclusion Criteria

1) Spinal cord injury based on clinical examinations or radiological findings, 2) Presence of any type of neurological diseases before the TBI or brain injury with non-traumatic causes such as; brain tumors, stroke, aneurysm and other brain vascular incidences, 3) Vegetative state or severe LOC, and unable to answer interview questions, 4) Not giving consent to enter the study for any reason.

### 3.3. Assessments

The demographic questionnaire was employed to record information about the patients’ age, gender, education level and GCS score to determine the severity of their HI in three categories; mild, moderate and severe. To study cognitive functioning, the mini–mental state examination (MMSE) was administered and to evaluate general health levels, the general health questionnaire (GHQ) - 28 items, was used. This questionnaire contains four subscales; physical symptoms, anxiety and insomnia, social dysfunction and depression. Their Cronbach’s alpha coefficients in Iranian TBI patients have been reported in a study by Rezaei *et al*. (15) as 0.81, 0.78, 0.91, and 0.86, respectively. The Glasgow Outcome Scale (GOS) was applied to assess general disability levels. Data were analyzed by an analysis of covariance (ANCOVA) and multivariate analysis of covariance (MANCOVA) adjusted for the effect of confounding variables (ie, age, gender, GOS, and level of education) to show the main effects of neurosurgical treatment and severity of head injury variables respectively. Fisher’s exact test and a chi square (χ ^2^) test were also utilized to determine the difference between categorical variables of the two groups. The results were considered as significant at *P* < 0.05.

## 4. Results 

Overall, 206 patients (166 males and 40 females) participated in this study. Mean age of the patients was 36.90 ± 16.95 (18 - 85 y). Table 1 and Table 2 show the descriptive indices (Mean ± SD) of cognitive functioning (MMSE) and general health (GHQ-28) variables (including; physical symptoms, anxiety and insomnia, social dysfunction, and depression) divided into; ‘with neurosurgery’ or ‘without neurosurgery’ and the severity of head injury (mild, moderate and severe). Of course, incomplete questionnaires were excluded from the statistical analysis.

**Table 1.** Descriptive Indices of Cognitive Functioning and General Health Based on Patients 'With Neurosurgery' or 'Without Neurosurgery'.

Neurosurgery Status	Mean ± SD	No.
**MMSE[Table-fn fn668]**		
No	23.09 ± 4.72	146
Yes	21.90 ± 7.10	60
Total	22.74 ± 5.52	206
**Physical symptoms**		
No	13.55 ± 4.17	13
Yes	11.01 ± 4.98	51
Total	12.86 ± 4.54	188
**Anxiety**		
No	12.73 ± 4.75	137
Yes	11.39 ± 4.70	51
Total	12.37 ± 4.76	188
**Social dysfunction**		
No	14.68 ± 4.58	137
Yes	13.14 ± 4.71	51
Total	14.26 ± 4.65	188
**Depression**		
No	7.50 ± 5.35	137
Yes	6.25 ± 5.78	51
Total	7.16 ± 5.48	188
**Total GHQ-28 score**		
No	48.74 ± 15.03	137
Yes	41.80 ± 16.51	51
Total	46.86 ± 15.71	188

Abbreviations: GHQ-28, general health questionnaire – 28 items; MMSE; mini–mental state examination

**Table 2.** Descriptive Indices of Cognitive Function and General Health Based on Severity of Head Injury

Severity of HI	Mean ± SD	No.
**MMSE[Table-fn fn667]**		
Mild	23.76 ± 4.64	147
Moderate	20.25 ± 5.93	27
Severe	19.15 ± 7.42	27
Total	22.68 ± 5.55	201
**Physical symptoms**		
Mild	13.47 ± 4.37	141
Moderate	11.96 ± 4.30	24
Severe	9.42 ± 4.67	19
Total	12.85 ± 4.55	184
**Anxiety**		
Mild	12.72 ± 4.82	141
Moderate	12.08 ± 4.04	24
Severe	10.00 ± 4.55	19
Total	12.35 ± 4.74	184
**Social dysfunction**		
Mild	14.33 ± 4.63	141
Moderate	13.83 ± 4.03	24
Severe	13.63 ± 5.14	19
Total	14.19 ± 4.59	184
**Depression**		
Mild	6.87 ± 5.27	141
Moderate	9.25 ± 5.34	24
Severe	6.47 ± 6.58	19
Total	7.14 ± 5.46	184
**Total GHQ**		
Mild	47.63 ± 15.67	141
Moderate	47.12 ± 13.41	24
Severe	39.52 ± 16.71	19
Total	46.73 ± 15.62	184

Abbreviations: GHQ, general health questionnaire; HI, head injury; MMSE, mini–mental state examination

In order to examine the effects of neurosurgical treatment and the severity of head injury on scores of cognitive functioning, an ANCOVA was used, the results of which are displayed in Table 3.

**Table 3.** ANCOVA for Effects of Neurosurgery Status and Severity of Head Injury on MMSE Scores

	df[Table-fn fn669]	SS	MS	F	*P* value
**Neurosurgery (A)**	1	0.002	0.002	0.001	0.99
**HI severity (B)**	2	486.48	243.24	8.65	0.0001
**Neurosurgery and HI severity (A×B)**	2	6.50	3.25	015	0.89
**Within group**	195	5486.23	28.13		

Abbreviations: F, F ratio; HI, head injury; MS, mean squares; SS, sum of squares

Based on the results of Table 3, only the main effect of head injury severity on the linear combination of MMSE scores (cognitive functioning), was significant [F (2, 195) = 8.65, *P* = 0.001], however, the effect of neurosurgery and its interaction (A×B) was not totally significant. Based on average numbers in Table 2, the effect of this factor demonstrated that patients with a more severe head injury compared to those with a mild injury had significantly poorer cognitive functioning. A MANCOVA with neurosurgical treatment, severity of head injury and their interaction as independent factors was also computed. The overall model was only significant in the severity of head injury. Wilks’ lambda = 0.89, [F (10, 348) = 1.99, *P* = 0.033]. To determine differences between the groups in terms of general health aspects, a MANCOVA was computed with age, gender, GOS, and level of education, as covariates. The results are illustrated in Table 4.

**Table 4.** MANCOVA for Effects of Neurosurgery Status and Severity of Head Injury on General Health Aspects

Dependent Variables	df	SS	MS	F	*P* value
**Neurosurgery[Table-fn fn670]**					
Physical symptom	1	0.97	0.97	0.05	0.819
Anxiety	1	15.43	15.43	0.72	0.398
Social dysfunction	1	10.180	10.180	0.50	0.479
Depression	1	0.241	0.241	0.008	0.927
Total GHQ-28 score	1	37.52	37.52	0.17	0.685
**HI severity**					
Physical symptom	2	207.76	103.88	5.62	0.004
**Anxiety**	2	135.10	67.55	3.14	0.046
Social dysfunction	2	12.04	6.02	0.30	0.743
Depression	2	89.61	44.81	1.56	0.214
Total GHQ-28 score	2	1157.21	578.61	2.55	0.081
**Neurosurgery and HI severity**					
Physical symptom	2	109.81	54.91	2.97	0.054
Anxiety	2	154.25	77.12	3.58	0.030
Social dysfunction	2	188.57	94.29	4.65	0.011
Depression	2	165.93	82.97	2.88	0.059
Total GHQ-28 score	2	2398.50	1199.25	5.29	0.006
**Within group**					
Physical symptom	178	3290.01	18.48		
Anxiety	178	3833.23	21.53		
Social dysfunction	178	3606.10	20.26		
Depression	178	5123.16	28.78		
Total GHQ-28 score	178	4035.35	226.67		

Abbreviations: F: frequency;GHQ-28: general health Questionnaire – 28 items; HI, head injury; MS, mean squares; SS, sum of squares

Based on Table 4, and consistent with Wilks’ lambda test, the effect of neurosurgical treatment on general health aspects was not significant. When we applied the Bonferroni correction, only the difference between the separate groups of head injury severity in terms of the physical symptoms [F (2, 178) = 5.62, *P* = 0.004] was truly significant.

**Figure 1.**
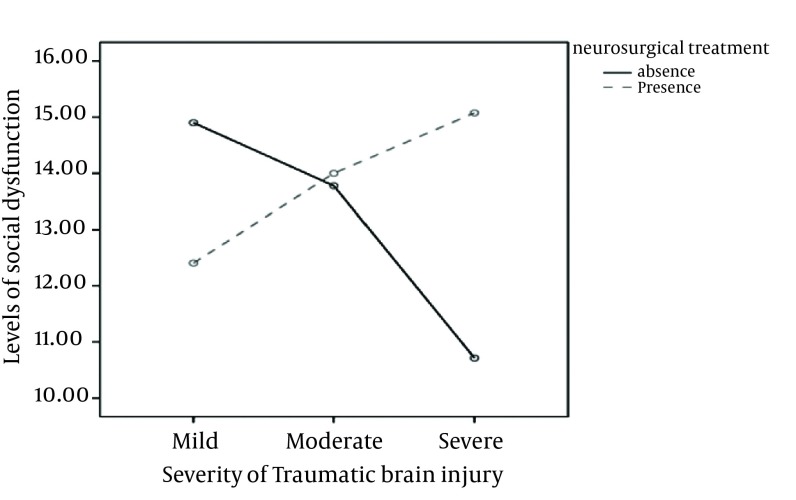
Interactive Effect of Neurosurgical Treatment and Severity of Head Injury on Social Dysfunction

**Figure 2.**
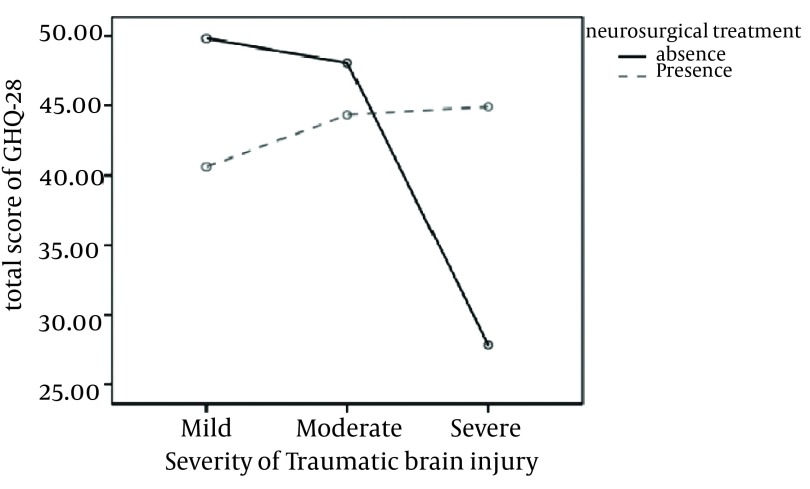
Interactive Effect of Neurosurgical Treatment and Severity of Head Injury on General Health Index (Total Score of GHQ-28)

Although in a multivariate Wilks’ lambda test, it was shown that overall the interaction of neurosurgery and head injury severity variables were not significant, but in a MANCOVA it was revealed that the interaction effect of these two variables using a Bonferroni correction on the aspects of social dysfunction [F (2, 178) = 4.65, *P* = 0.011] and total scores of the GHQ-28 [F (2, 178) = 5.29, *P* = 0.006] test was significant. A MANCOVA followed by a Gabriel post-hoc test considered in Table 2, showed that patients with a mild severity of head injury compared to severely injured ones, had a significantly higher level of physical symptoms (*P* < 0.0001) and when the interaction effects of these factors were calculated, not having neurosurgical treatment in severe head injured patients was accompanied with significantly lower levels of social dysfunction (Figure 1) and better general health (Figure 2).

After three months of follow-up, by reminding them via a phone call, only 155 patients with an average of 145 ± 53 days (about four months) referred for a psychiatric examination. Each of these patients was examined through a structured clinical interview based on DSM-IV diagnostic criteria, to determine the incidence of mental disorders after a traumatic brain injury.

Table 5 shows, no significant difference between the two groups with or without mental disorders in terms of neurosurgical treatment (Fisher’s exact test, *P* = 0.69). The results of a chi-square test showed that there is a significant difference between the two groups of patients with and without mental disorders in terms of different levels of head injury ( χ2 = 8.85, df = 2 , *P* = 0.008). This means that patients with a more severe head injury, fourmonths after TBI, were more likely to be diagnosed with a mental disorder.

**Table 5.** Comparing Patients With and Without Mental Disorders Regarding Neurosurgery Status and Severity of Head Injury

	Positive, No.(%)[Table-fn fn671]	Negative, No. (%)	Total, No. (%)	*P* value
**Neurosurgery**				0.69
Yes	37 (77.1)	72 (73.5)	109 (74.7)	
No	11 (22.9)	26 (26.5)	37 (25.3)	
**HI severity**				χ^2^ *P* = 0.008
Mild	64 (67.4)	31 (32.6)	95 (65.7)	
Moderate	23 (82.1)	5 (17.9)	28 (19.2)	
Severe	22 (95.7)	1 (4.3)	23 (15.1)	

Abbreviation: HI, head injury

## 5. Discussion

The present research aimed at studying the effect of neurosurgical treatment and severity of head injury on cognitive functioning, general health and incidence of mental disorders in TBI patients. The authors expected to observe more cognitive impairments and psychological symptoms in TBI patients following neurosurgical treatment and manipulation of brain tissue to amend the injuries due to penetrating damages, skull fractures, managing all types of bleeding, and reducing intracranial pressure. Hence, as results of the present study have revealed, neurosurgical treatment had no negative effect on cognitive status and general health in TBI patients. Even after four months of follow-up, it was revealed that whether they had neurosurgical treatment or not, had no negative impact on the incidence of mental disorders following TBI. After reviewing the previous research literature, we did not find any study testing this hypothesis; so, we can cautiously state that at times when neurosurgical treatment is deemed to be necessary, there should be no concern about the procedure worsening cognitive and psychiatric outcomes in TBI patients. Results also indicate that greater head injury severity is accompanied with poorer cognitive functioning in TBI patients. The devastating effects of a more severe head injury not only include cognitive impairments, but these may also bring about physical problems and disabilities. It was, however, found that even with a reduction of physical damage and medical problems, cognitive impairment may still last for years after a brain injury (23, 24). No established pharmacological strategies have been offered to resolve cognitive impairment in TBI victims, but other interventions such as cognitive rehabilitation after TBI have gained a special place in clinical perspectives and empirical investigations (25, 26). Cognitive rehabilitation is useful for the treatment of memory impairments following a TBI. Cognitive rehabilitation may also be useful for the treatment of impaired attention, interpersonal communication skills and executive functioning following a TBI. This form of treatment is most useful for patients with mild to moderate cognitive impairments, and may be particularly useful for those who are still relatively functionally independent and motivated to engage in and rehearse these strategies (4). This study also demonstrated that patients with a less severe head injury than those with more severe degrees, reported higher levels of physical symptoms. This finding seemed a little odd at first; since we expected a reduction of general health indices in the GHQ-28 with an increase in the severity of head injury, however, the result turned out to be vice versa. To justify this finding, it can be stated that self-report measures may not be reliable and valid in TBI survivors with impaired insight into their disabilities and mental and physical symptoms (27). Decreased self-awareness is common after a TBI (28), therefore in self-report measures a lack of awareness may lead to an under-diagnosis of psychiatric syndromes. In this case, patients with a more severe TBI than those with a milder head injury fail to recognize and report cognitive, social and emotional impairments. In addition, Fan *et al*. (29) have also pointed out that a secondary benefit can also affect the accuracy of self-evaluation tools. Findings of this study showed that the interaction of neurosurgical treatment and head injury (A × B) is significant on physical symptoms and also on total scores of GHQ-28. According to Table 1, similar to the above mentioned finding, we concluded that general health indices in patients with more severe head injury have a greater drop. In this case, this is correct about the interpretation of the previous finding, but when the interactive effect of neurosurgical treatment and severity of head injury are entered together into the multivariate analysis, the components of general health in patients with any degree of head injury improve substantially (Table 4). Thus, we can be hopeful about the corrective role of neurosurgical treatment in improving general health aspects of those patients who need neurosurgery, particularly in the area of physical symptoms. The four month follow-up of TBI patients showed a significant difference between patients with and without mental disorders in terms of the severity of head injury, which means more mental disorders were diagnosed in patients with a more severe TBI (Table 5). This finding is consistent with results from the Fan *et al*. study (29) and Smith’s point of view (17) which is based on the incidence of mental disorders and other psycho-neurological problems increasing with the severity of brain damage. Regarding studies using correlational methods between TBI severity and its psychopathological outcomes, findings of prior studies confirm the results of this study. For example, Van Reekum *et al*. (30) considered the severity of TBI among risk factors in the incidence of mental disorders. Max *et al*. (19) have also concluded that the severity of TBI is the only variable that predicts personality changes after a TBI. Verma *et al*. (31) and Mainio *et al*. (21) found that the severity of a TBI was related to some evaluations of sleep disruption and the incidence of suicidal behavior respectively. It can be stated that a more severe head injury due to; physical, neurological, and cognitive disabilities, consequently produces more social limitations thus resulting in the incidence of mental disorders in these patients. The main limitation of this study was that the MMSE test was used to demonstrate the level of cognitive functioning. Given the fact that neurosurgery was performed in different parts of the brain and each of these areas is responsible for various cognitive functions, a neuropsychological test battery should have been used to gather more accurate measurements of cognitive functioning. Overall, the results of this research endorse the role of neurosurgical treatment in cognitive and neuropsychiatric outcomes of TBI patients. It has also been clarified that head injury severity is the most important factor in the deterioration of cognitive functioning and general health, and it even plays a key role in the incidence of mental disorders four months after a TBI. These results were achieved while the role of confounding variables such as; age, gender, education level and GOS scores were controlled. It is suggested that neuropsychological rehabilitation programs be used to manage cognitive impairments in patients with TBI. Furthermore to improve symptoms of psychopathology in addition to psychiatric drugs, diverse types of psychotherapy: supportive, individual, cognitive-behavioral, group and family therapy can be used. It is also vital that in cases in which the patient has a severe head injury, along with stable cognitive impairments, that both patients and families are helped to adapt to the stable disabilities resulting from a TBI.
